# Plasma single-stranded DNA autoantibodies in the diagnosis of Hirschsprung’s disease

**DOI:** 10.3389/fmed.2022.1013785

**Published:** 2022-11-07

**Authors:** Bingtong Wang, Yongxuan Yao, Wenlin Fang, Yanqing Liu, Wei Zhong, Ye He, Yulu Lai, Qiuming He, Yun Zhu, Chaoting Lan

**Affiliations:** ^1^Guangzhou Women and Children’s Medical Center, Guangdong Provincial Clinical Research Center for Child Health, Guangzhou Medical University, Guangzhou, China; ^2^School of Medicine, South China University of Technology, Guangzhou, China; ^3^Southern Medical University, Guangzhou, China

**Keywords:** ENS, HSCR, ssDNA antibodies, early diagnosis of HSCR, ELISA

## Abstract

**Background:**

Hirschsprung’s disease (HSCR) is a neonatal enteric nervous system (ENS) disease characterized by congenital enteric ganglion cell loss. The only treatment is aganglionic bowel segment resection and innervated bowel segment reconstruction. Delayed diagnosis and treatment cause postoperative complications such as intractable constipation and enterocolitis. Existing preoperative HSCR diagnostic methods have shortcomings such as false positives, radiation and invasiveness.

**Methods:**

We used the robust linear model (RLM) for normalization and the M statistic for screening plasma human autoimmune antigen microarrays and quantitatively assessed single-stranded DNA (ssDNA) antibody levels with enzyme-linked immunosorbent assay (ELISA).

**Results:**

The autoimmune antigen microarray revealed that autoantibodies were higher in HSCR plasma than in disease control (DC) and healthy control (HC) plasma. ssDNA antibodies in HSCR plasma were significantly higher than those in DC and HC plasma. Quantitative ssDNA antibody level detection in plasma by ELISA showed that HSCR (*n* = 32) was 1.3- and 1.7-fold higher than DC (*n* = 14) and HC (*n* = 25), respectively. ssDNA antibodies distinguished HSCR from non-HSCR (HC and DC), achieving an area under the curve (AUC) of 0.917 (95% CI, 0.8550–0.9784), with a sensitivity of 96.99% and a specificity of 74.63%.

**Conclusion:**

ssDNA antibodies in plasma can serve as a diagnostic biomarker for HSCR in the clinic.

## Introduction

Hirschsprung’s disease (HSCR) is a birth defect in which intestinal nerves in children develop abnormally and is characterized by the absence of ganglion cells in the distal intestine. The pathological mechanism is the disturbance of the migration and differentiation of enteric neural crest cells into enteric neurons, resulting in persistent spasm due to a lack of enteric nerves (1, 2). HSCR is a common congenital disorder of the intestinal tract in children, and the worldwide incidence is approximately 1 in 5,000 live births, with a male to female ratio of 4:1 (3, 4). The early clinical symptoms of HSCR are abdominal distension, diarrhea and vomiting (5, 6). To date, surgical resection of the aganglionic bowel segment, with reconstruction of the normally innervated intestine, is the only definitive treatment for HSCR. Surgical management for HSCR has significantly improved symptoms; however, approximately one-third of patients with HSCR still develop postoperative complications such as enteritis and refractory constipation (6–9).

The diagnosis of HSCR relies on interventional biopsy, including surgical full-thickness rectal biopsy or rectal suction biopsy (RSB), by aspirating rectal mucosal tissue (10). In addition, contrast enema (CE), which shows the spastic segment, the dilated segment and the transition segment using X-rays, is the most commonly used method to assist in the diagnosis of HSCR. Anorectal manometry (ARM), which determines abnormal innervation of the enteric nerve by detecting the inhibitory reflex of the internal anorectal sphincter, is non-invasive but not accurate in the independent diagnosis of HSCR. Additionally, the above diagnostic methods are associated with technical difficulty, radiation exposure, and invasiveness (11–13). In addition to the above limitations, the clinical symptoms of HSCR overlap with functional constipation, anal atresia, and intestinal strictures, making it difficult to differentiate it from these diseases (10). Clinical data show that early diagnosis of HSCR can obtain good prognosis and reduce the occurrence of complications (14). Various problems with the current diagnostic methods have prompted us to investigate more sensitive, efficient and non-invasive diagnostic methods.

Genetic factors explain only a small fraction of HSCR risk. Infections and the immune response have been reported to be involved in the pathogenesis of HSCR (15–17). Viral infection was reported to induce DNA breakage and produce DNA autoantibodies (18). Single-stranded DNA (ssDNA) antibodies can bind nuclear DNA to induce apoptosis; thus, ssDNA antibodies are sensitive markers for programmed cell death and drug-induced apoptosis (19, 20). ssDNA antibodies are specifically activated and amplified in the serum of patients with systemic lupus erythematosus (SLE), particularly drug-induced SLE (21–23). Therefore, ssDNA antibodies in serum can be used for the clinical diagnosis of drug-induced SLE. A positive correlation between the level of serum ssDNA antibodies and the severity of linear scleroderma (LS) makes ssDNA antibodies the diagnostic marker of disease severity in LS (24, 25). In addition, the cross-reaction of ssDNA antibodies and α-actinin antibodies in the serum of autoimmune hepatitis (AIH) patients can be used to monitor AIH disease activity (26).

In this study, we first screened activated autoantibodies using an autoimmune antigen microarray and found that ssDNA antibodies were increased in the plasma of HSCR patients. We speculated that elevated ssDNA antibody concentrations in plasma are highly diagnostic for HSCR. To test this hypothesis, we increased the sample size to quantify ssDNA antibody levels in the plasma in independent samples. The results showed that ssDNA antibodies could distinguish HSCR from non-HSCR (HC and DC), achieving an area under the curve (AUC) of 0.917 (95% CI, 0.8550–0.9784) with a sensitivity of 96.99% and a specificity of 74.63%. Herein, we found that ssDNA antibodies can serve as novel biomarkers for HSCR diagnosis.

## Materials and methods

### Study subjects

The plasma analyzed in this study were collected from 86 children in Guangzhou Women’s and Children’s Medical Center who were aged from 3 months to 3 years, with a male: female ratio of 4:1. Plasma samples were divided into the HSCR group, the disease control (anal atresia and intestinal stricture, DC) group and the healthy control (HC) group. The disease and control groups were age and sex matched. This study was approved by the Institutional Review Committee of Guangzhou Women and Children’s Medical Center (No. 2018052406). Written consent was obtained from the participant’s parents or their legal guardian.

### Preparation of plasma

Fasting peripheral blood samples (3 ml/participant) were collected from participants using 4 ml EDTA vacuum tubes. For the HSCR group and DC group, blood was collected during the operation. The plasma of the HC group was collected from the plasma remaining from the health check-up. The blood collected in EDTA vacuum tubes was processed within 2 h. The plasma was obtained by centrifugation at 1,500 × rpm at 4°C for 20 min and then centrifuged again at 3,000 × rpm at 4°C for 15 min. The collected plasma was stored at-80°C.

### Plasma ssDNA measurement

We randomly selected 5 plasma samples from each of three groups, the HSCR group, the DC group and the HC group, and then performed human autoimmune antigen microarray screening. Yijin Biotechnology Co., Ltd., performed IgG testing, including testing of more than 100 autoimmune antibodies. The level of ssDNA antibodies in the plasma was detected by ELISA using a ssDNA antibody ELISA Kit (Shanghai Zhenke Biotechnology Co., Ltd., ZK-1319). The person in charge of the test was blinded to the results and the final diagnosis. Three technical replicates were performed for each sample, and the mean value was recorded.

### Statistical analysis

Data were statistically analyzed using R and GraphPad Prism 8.0 (GraphPad Software Inc., CA, USA). We used the robust-linear-mode (RLM) method for normalization and M-statistics for screening (27) and then performed cluster analysis on the screening results of the human autoimmune antigen microarray. Quantitative variables were analyzed using a *t*-test. A receiver operating characteristic (ROC) curve was used to calculate the cut-off value of ssDNA antibodies and the AUC to analyze the diagnostic value of ssDNA antibodies. Sensitivity and specificity were used to assess the diagnostic accuracy of the markers. The AUCs were between 0.1 and 1. The closer the AUC is to 1, the more reliable the antibodies are as a diagnostic marker. *P* < 0.05 indicated statistical significance, and all *P*-values are from two-sided tests.

## Results

### Characteristics of the subjects

To rule out the possibility that ssDNA antibodies could serve as a biomarker for HSCR diagnosis. We first included 86 subjects (Table 1) for testing. Among these subjects, HSCR (*n* = 37) was diagnosed by postoperative pathological biopsy. The DC (*n* = 19) was diagnosed by clinical manifestations and auxiliary examinations: (1) the clinical manifestations were the absence of meconium discharge after birth and skin covering the anal area; (2) auxiliary examination using an inverted lateral X-ray film that demonstrated that the end of the rectum was at or slightly below the pubococcygeal line. HC (*n* = 30) refers to healthy children between the ages of one and three who attended the center for a health check-up.

**TABLE 1:** Sample information of the research subjects.

Characteristic	Cases	Controls		*P* ^a^
			
N	HSCR^b^ (37)	DC^b^ (19)	HC^b^(30)	–
Gender (Female; Male)	9/28	5/14	8/22	<0.05
Age (month)^c^ (≤5; >5)	12.8 ± 9.93	8.70 ± 4.44	14.61 ± 5.52	<0.05

Two-tailed χ^2^-test of the distribution between HSCR cases and controls; HSCR, Hirschsprung’s disease; DC, disease control (including anal atresia and intestinal stricture); HC, healthy control; Age (month) of onset for cases: (mean ± SD). SD, Standard deviation.

### Screening of human autoimmune antigen microarray and analysis of differentially expressed autoantibodies

**FIGURE 1:**
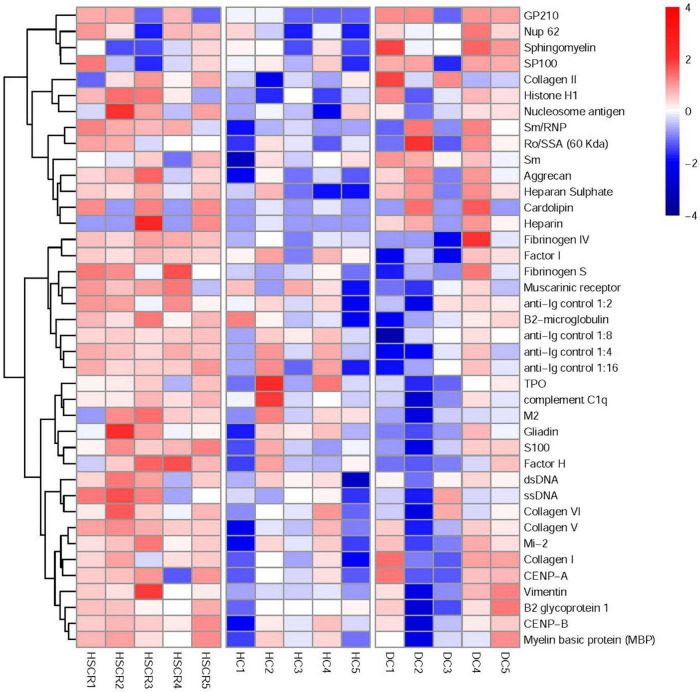
Screening of human autoimmune antigen microarray and analysis of differential autoantibodies. The robust linear model (RLM) method was used for normalization and M statistics for screening, and then the screening results of the human autoimmune antigen microarray were subjected to cluster analysis.

We further analyzed the screening results of the human autoimmune antigen microarray using the RLM method and M statistical analysis. As shown in Figure 1, the cluster analysis results indicated that the levels of autoantibodies were significantly different among HSCR, DC and HC plasma. In particular, compared to the DC and HC plasma, the level of ssDNA antibodies in HSCR plasma was observably increased (*p* = 0.019). These results demonstrated that ssDNA antibodies may correlate with HSCR.

### ssDNA antibody expression in Hirschsprung’s disease, disease control and healthy control plasma

**FIGURE 2:**
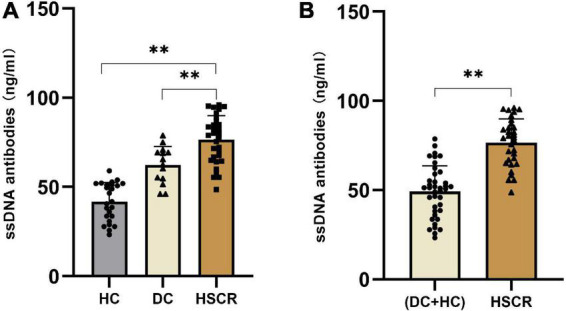
The ssDNA antibodies increased in HSCR plasma. **(A)** The level of ssDNA antibodies in HSCR group were compared with the DC and the HC group, respectively. **(B)** The level of ssDNA antibodies in HSCR group were compared with the DC and HC combined control group. “**P < 0.01” indicates statistical significance, and all *P*-values were tested by two-sided tests.

To quantify ssDNA antibody expression in HSCR, DC and HC plasma, we detected ssDNA antibodies by ELISA. The results showed that, compared to DC and HC plasma, the ssDNA antibodies were notably increased by approximately 1.3- and 1.7-fold, respectively, in HSCR plasma (Figure 2A). As expected, ssDNA antibodies in HSCR plasma were approximately 1.5-fold higher than those in the DC and HC combined control group (Figure 2B).

### The ssDNA antibodies in plasma can serve as a diagnostic marker for Hirschsprung’s disease

**FIGURE 3:**
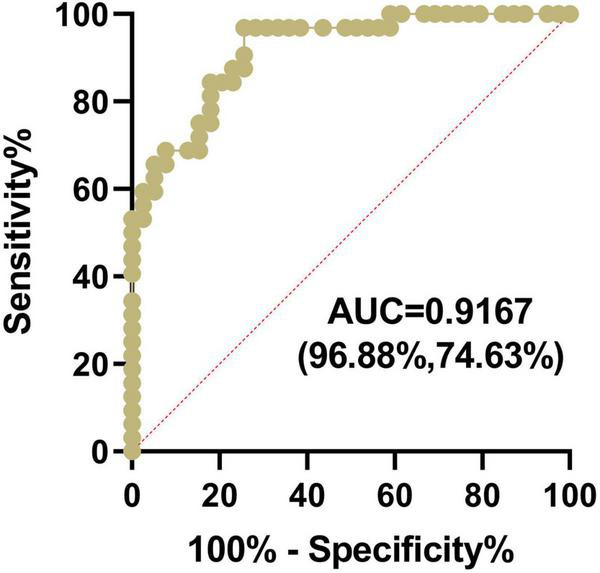
The ssDNA antibodies in plasma can serve as a diagnostic marker for HSCR. The Plasma ssDNA antibodies from the HSCR and control group were measured by ELISA, and the sensitivity and specificity of ssDNA antibodies for HSCR diagnosis were subjected to AUC analysis.

We further investigated the sensitivity and specificity of ssDNA antibodies for HSCR diagnosis. The results showed that the AUC of the ssDNA antibodies in HSCR was 0.9167, and the optimal limit corresponded to a sensitivity of 74.63% and a specificity of 96.88% (Figure 3). This result suggested that ssDNA antibodies in plasma can serve as a biomarker for HSCR diagnosis.

## Discussion

HSCR is an enteric nervous system (ENS) disorder in children. It is crucial to identify sensitive and non-invasive diagnostic markers for early HSCR diagnosis, which will help significantly improve the quality of life and overall survival of children with HSCR after surgery. At present, the clinical methods for the preoperative diagnosis of HSCR include RSB, CE and RAM. RSB was the most effective method for HSCR diagnosis in children older than 39 days, with an average sensitivity of 88%, but sensitivity dropped to 50% in children under 39 days compared to older children, as continuous dynamic development of the ENS after birth leads to inaccuracy of RSB in younger children with HSCR (28). Moreover, SRB is an invasive method that may increase the likelihood of intestinal bleeding and intestinal perforation (29). CE and RAM are auxiliary diagnostic methods for HSCR. CE is radioactive with a sensitivity of 70–76% and a specificity of 83–97% (12, 30). The sensitivity and specificity of RAM for the diagnosis of HSCR were 83% and 93%, respectively (30). It is generally believed that the RAM method suffers from an excessive false-positive rate in infants younger than 14 weeks due to immaturity of the neural internal sphincter reflex (31, 32). The above methods have limitations, such as invasiveness, radiation exposure and false positives. In our study, plasma ssDNA antibodies had the highest sensitivity for diagnosing HSCR, reaching 96.88% (vs. 93, 83, and 70–76% for RSB, RAM, and CE, respectively). These results support the application of ssDNA antibodies in plasma as a non-invasive diagnostic marker for HSCR.

Some breakthroughs have been made in the current research on the invasiveness and radiation problems associated with preoperative HSCR diagnostic methods. Hydrocolonic sonography (HS) was recently used to diagnose HSCR, with a sensitivity and specificity of 89.8 and 96.3%, respectively (33). A previous study by Tang et al. (34) showed that the AUC of serum microRNAs (miRNAs) for the diagnosis of HSCR was 0.895, with a sensitivity and specificity of 80 and 95%, respectively. miRNAs are currently known as novel biomarkers for the non-invasive early screening of HSCR (11, 34, 35). The AUC of ssDNA antibodies in our current study was 0.917 (0.895 vs. miRNAs), with a sensitivity of 96.88% (80% vs. miRNAs) and a specificity of 74.63% (95% vs. miRNAs). The above comparisons show that the ssDNA antibodies have better AUC and sensitivity in diagnosing HSCR.

HSCR and DC have overlapping clinical symptoms and are difficult to differentiate based on existing early diagnosis methods. We found that plasma ssDNA antibodies were 1.3- and 1.7-fold higher in the HSCR group than in the DC and HC groups, respectively (Figure 2A). This difference was significant. Our preliminary analytical results suggested that ssDNA antibodies seem to be a specific biomarker for HSCR without interference from DC. In summary, we found that ssDNA antibodies in plasma can be used as biomarkers for the diagnosis of HSCR, with the advantages of simple operation, high throughput, and low cost. Moreover, we found that ssDNA antibodies decreased in postsurgery HSCR patients compared with those in presurgery HSCR patients, probably due to the surgical resection of the diseased segment of the colon. Interestingly, ssDNA antibodies increased in postsurgery patients with Hirschsprung-Associated Enterocolitis (HAEC) compared with those in non-HAEC patients and was comparable to the presurgery level (Supplementary Figure 1). These results indicated that ssDNA antibodies would not only be applied as a marker of HSCR but also serve as a follow-up marker of postsurgery HAEC.

Nevertheless, our study has some limitations. First, this study was conducted in a single center, and the samples were mostly from southern China. To confirm that the application of a new biomarker is effective for the majority of the population, samples from different ethnicities and regions are needed. Second, this was a retrospective study, and a prospective study with long-term follow-up of clinical outcomes will provide stronger evidence for determining the diagnostic accuracy of a new biomarker (36). Finally, a novel diagnostic marker needs to have high sensitivity and specificity. The sensitivity of ssDNA antibodies in plasma to diagnose HSCR is as high as 96.88%, while the specificity is only 74.63%, which can be combined with highly specific auxiliary diagnostic methods such as CE. In brief, ssDNA antibodies may be used as plasma diagnostic markers for HSCR, providing an effective method for the clinical diagnosis of HSCR.

## Data availability statement

The original contributions presented in this study are included in the article/Supplementary material, further inquiries can be directed to the corresponding authors.

## Ethics statement

This study was approved by the Institutional Review Committee of Guangzhou Women and Children’s Medical Center (No. 2018052406). The written consent of the participant’s parents or their legal guardian has been obtained.

## Author contributions

CL, YZ, and WZ designed the experiment. YH, YLL, and QH queried and collected information about cases. BW, YH, WF, and YQL involved in collecting samples and conducting experiments. BW, CL, YZ, and YY analyzed the data from the experimental results. BW, CL, and YY wrote the manuscript. All authors read and approved the manuscript.

## Supplementary material

Supplementary Figure 1 Comparison of ssDNA antibodies in post-surgery HSCR patients.
