# Supplementary figures and images for: Plasma single-stranded DNA autoantibodies in the diagnosis of Hirschsprung’s disease
Source: Front Med (Lausanne). 2022 Nov 7;9:1013785. doi: 10.3389/fmed.2022.1013785 (PMC9676256; doi:10.3389/fmed.2022.1013785)

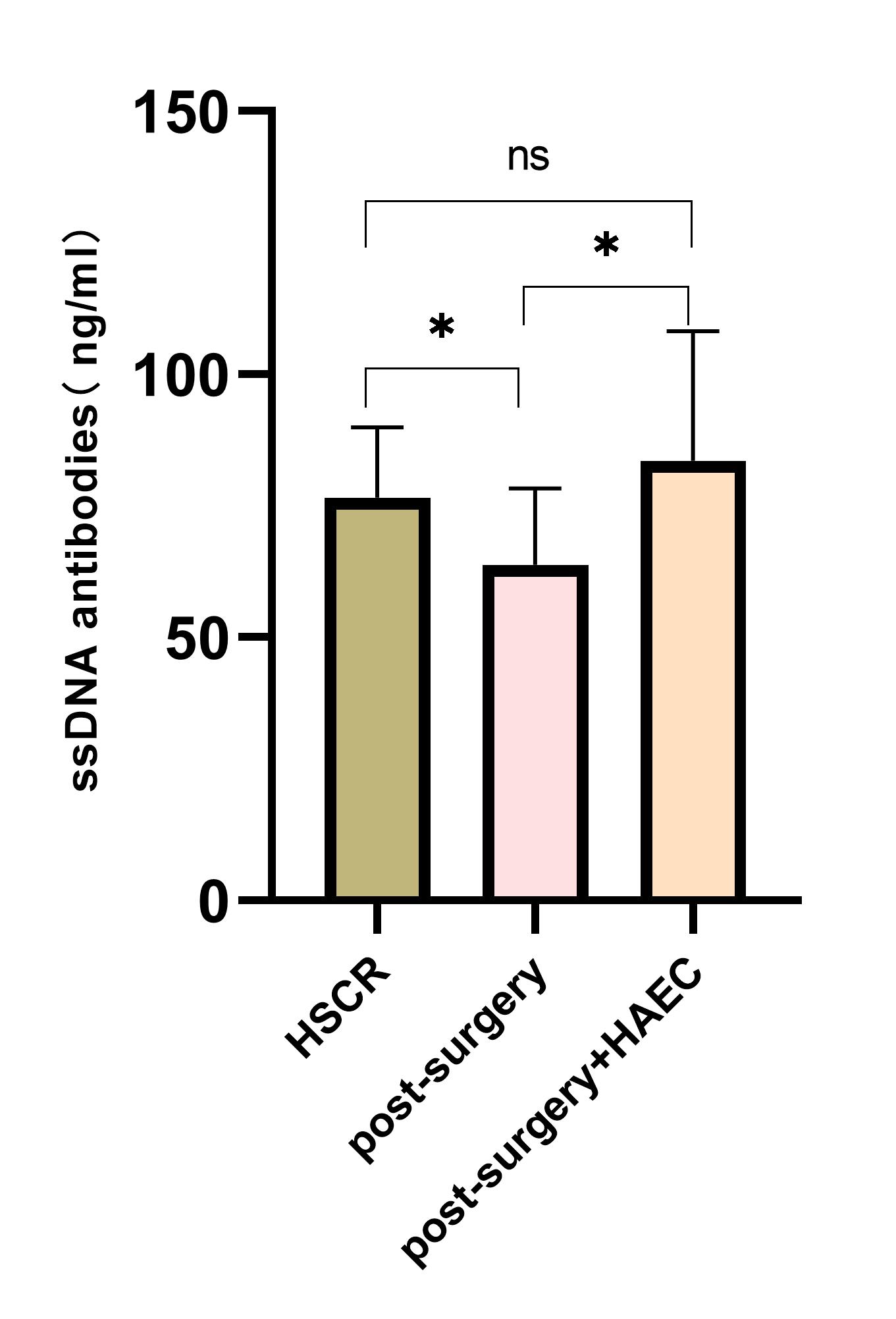

Supplement: Supplementary Figure 1 — Comparison of ssDNA antibodies in post-surgery HSCR patients. [file Image_1.JPEG]
